# Treatment of Blood Blister Aneurysms of the Internal Carotid Artery With Pipeline-Assisted Coil Embolization: A Single-Center Experience

**DOI:** 10.3389/fneur.2022.882108

**Published:** 2022-06-13

**Authors:** Peng Liu, Lunxin Liu, Changwei Zhang, Sen Lin, Ting Wang, Xiaodong Xie, Liangxue Zhou, Chaohua Wang

**Affiliations:** Department of Neurosurgery, West China Hospital of Sichuan University, Chengdu, China

**Keywords:** blood blister aneurysm, pipeline embolization device, internal carotid artery, coil embolization, subarachnoid hemorrhage

## Abstract

**Background:**

Blood blister aneurysm (BBA) is a complex and rare aneurysm that presents significant treatment challenges. The application of pipeline embolization device (PED)-assisted coiling in the treatment of ruptured BBA remains controversial. This study aimed to report on our experience and assess the safety and efficacy of this strategy.

**Methods:**

Between February 2019 and February 2021, 12 patients with ruptured BBAs underwent PED-assisted coil embolization. We collected detailed data about each patient, including demographic information, aneurysmal data, technical details, antiplatelet strategy, operation-related complications, and follow-up outcomes.

**Results:**

A total of 12 BBA patients were treated with single PED-assisted coil embolization. One patient experienced intraoperative rupture that was controlled by rapid coiling without clinical consequences. All the patients demonstrated complete occlusion on postoperative angiography. A total of three patients had postoperative complications: left hemiparesis, Broca's aphasia, and right hemiplegia due to vasospasm, and transient hemiparesis. Follow-up angiography revealed that all BBAs were completely occluded, except one with neck residue. All patients had favorable outcomes at discharge and the most recent clinical follow-up (mRS score ≤ 2).

**Conclusion:**

Endovascular treatment of BBAs of the internal carotid artery using PED-assisted coil embolization is a safe and effective strategy. This has contributed to the understanding of BBA therapy and provides a potentially optimal treatment option for this intractable lesion.

## Introduction

A blood blister aneurysm (BBA) is a rare type of aneurysm. It accounts for approximately 0.3–1% of intracranial aneurysms and 0.9–6.5% of ruptured aneurysms (1). It is characterized by thin-walled broad-based aneurysms that lack an identifiable neck and have a high risk of rupture (2). Unlike a saccular aneurysm, a BBA typically originates from the non-branching sections of the supraclinoid portion of the internal carotid artery (ICA) (3). This complex character poses significant challenges for treatment.

Although the efficacy of microsurgical treatment of BBA has been demonstrated, the endovascular intervention has gradually become the preferred alternative for BBA treatment, owing to its lower complication rate and better prognosis (4, 5). While there are numerous interventional techniques in the treatment of BBA, including various intracranial stents with or without coiling, endovascular ICA trapping, and a combination of other procedures, the invention of the flow diverter (FD) represents a milestone in the history of aneurysm treatment (6–8). The pipeline embolization device (PED), the most often used FD, promotes intra-aneurysmal occlusion by reconstructing the blood flow in the parent artery. At present, more reports have focused on the use of PED alone for BBA treatment. Complications, such as delayed occlusion, resulting in recurrence or possibly re-rupture, raise concerns and restrict its application (6, 7). Using PED combined with coil embolization may be a reasonable and feasible strategy. However, few studies have reported the effects of PED-assisted coil embolization in BBA treatment. Therefore, this study aimed to evaluate the safety and efficacy of this strategy, enhance our understanding of BBA therapy, and provide a potentially optimal treatment option for intractable lesions.

## Methods

### Data Sources and Variables

Between February 2019 and February 2021, 12 consecutive patients with subarachnoid hemorrhage (SAH) due to ruptured BBA of the ICA were treated with PED-assisted coil embolization at West China Hospital of Sichuan University. Three-dimensional angiography was performed to confirm all BBAs. PED was used to treat BBA only in the absence of hydrocephalus, severe vasospasm, cerebral infarction, and the Hunt and Hess grade lower than III. Detailed information was extracted from the medical records of each patient, including patient demographics, aneurysmal data, technical details, antiplatelet strategies, operation-related complications, and follow-up outcomes. The safety and efficacy of this treatment modality were also evaluated. Safety was assessed based on perioperative complications and follow-up neurological function. Postoperative and follow-up occlusions were considered the criteria for efficacy. This study was approved by the Ethics Committee of the West China Hospital of Sichuan University.

### Technical Details

The procedure was performed under general anesthesia, and systemic heparinization was administrated before the procedure. A triaxial guide-catheter system, including 9F short femoral sheath (Terumo, Tokyo, JPN), 7F 90 cm long sheath (COOK, Indiana, USA), and 5F 115 cm Navien (Medtronic, California, USA) intermediate catheter, were introduced into the left or right femoral artery. The 7F sheath was inserted into the C1 segments of the ICAs using a 125 cm vertebral catheter (Terumo, Tokyo, JPN). The Navien intermediate catheter was advanced into the C2 segment to complete the parent artery angiography and three-dimensional (3D) rotational angiography. To choose a suitable PED (Medtronic, California, USA) and coil size, the diameters of the proximal and distal parts of the parent vessel, aneurysm, and neck were measured. The Marksman (Medtronic, California, CA) microcatheter was navigated to the M2 segment through the Navien catheter. After that, a steam-shaped Echelon-10 microcatheter (Medtronic, California, USA) was delivered to the aneurysmal sac *via* the 7F long sheath located next to the Navien catheter. The Echelon-10 microcatheter was removed from the aneurysm sac to the parent artery, after confirming whether it has the suitable shape to navigate to the aneurysm sac easily. A suitable PED was then semi-deployed to the distal of the aneurysm neck, subsequently, the Echelon-10 microcatheter was navigated to the aneurysm sac again, and some loops of coils were inserted into the aneurysm sac. After the aneurysm cavity was completely embolized, the PED was fully deployed. Digital subtraction angiography (DSA) was performed immediately after PED placement to evaluate the degree of embolization, stent positioning, and patency of the branch and parent arteries. Vasospasms and operation-related complications were recorded.

### Perioperative Management

After admission, all patients received specialist care and systemic treatment to withstand the surgery. Tirofiban (0.2 μg/kg/min) was administered immediately following successful stent placement. One day after the procedure, tirofiban was discontinued after a loading dose of 225 mg clopidogrel and 300 mg aspirin for 6 h. All patients continued aspirin 100 mg/day for 6 months and clopidogrel 75 mg/day for at least 3 months, beginning the next day.

### Follow-Up

We conducted clinical and imaging follow-ups for all patients. The modified Rankin score (mRS) was used to assess neurological outcomes at discharge and at the most recent clinical follow-up. An mRS score of 0–2 indicated that the patient had a positive neurological prognosis. Imaging follow-up was performed 3–6 months postoperatively using DSA to assess aneurysmal occlusion, which was quantified using the simplified Reymond grade (grade 1, complete occlusion; grade 2, neck remnant; grade 3, residual sac) (8). Additionally, the patency of the branch and parent vessels, degree of embolization, and position of the PED were documented. Two neurointerventional specialists assessed these findings.

## Results

This study involved 12 patients (six women and six men) who had acute SAH due to BBAs. The mean age of the patients was 44.2 years (range, 34–56 years). On admission, nine patients had a Hunt and Hess classification of II, and III in three patients. The Fisher grade was 3 in six patients, 2 in five patients, and 1 in one patient. The BBAs were located at the C6 segment of the ICA in five patients and C7 in seven patients, ranging from 1.4 × 1.3 mm to 5.9 × 3.9 mm. The average duration from disease onset to treatment was 5.8 days (range, 2–16 days). The baseline clinical and imaging characteristics are shown in Table 1.

**Table 1:** Baseline clinical and imaging characteristics.

**Case no**.	**Sex/Years**	**Presentation**	**Other disease**	**H&H**	**FGS**	**Aneurysm location**	**Aneurysm size (mm)**	**Aneurysm neck (mm)**	**DT (day)**
1	M/34	SAH	N	2	2	R-C7	3.3 × 2.6	3.5	8
2	F/47	SAH	asthma	2	3	L-C7	4.3 × 3.6	4.3	2
3	F/47	SAH	N	2	3	L-C6	1.6 × 1.4	1.9	4
4	F/46	SAH	hypertension	3	3	L-C7	3.7 × 3.4	7.2	5
5	F/49	SAH	N	2	3	R-C7	1.4 × 1.3	1.3	16
6	M/53	SAH	N	2	1	R-C6	3.5 × 2.0	3.5	5
7	F/36	SAH	pneumonia	2	2	R-C7	5.9 × 3.9	5.3	4
8	M/44	SAH	N	2	2	L-C7	3.4 × 3.0	3.1	3
9	M/56	SAH	N	3	2	R-C6	2.9 × 2.1	3.1	4
10	F/39	SAH	hypertension	3	3	L-C7	3.4 × 3.2	3.7	5
11	M/43	SAH	N	2	2	L-C6	4.7 × 3.3	5.4	7
12	M/37	SAH	N	2	3	L-C6	5.3 × 4.2	6.1	6

*F, female; M, male; H&H, Hunt and Hess grade; FGS, Fisher grade scale; SAH, subarachnoid hemorrhage; DT, Diagnosis to treatment*.

All enrolled patients were treated with single PED-assisted coil embolization. Of the 12 cases, complete occlusion (Raymond grade I) was confirmed using immediate angiography after the operation. Intraoperative angiography revealed vasospasm in five patients. None of the patients in our study underwent extraventricular drainage or other surgical procedures. None of the patients experienced intraoperative complications, except for one patient (Patient 1) who experienced an aneurysm rupture that was successfully treated with urgent dense coiling. Three patients experienced adverse post-procedural complications related to vasospasm. Patient 1 developed left limb hemiplegia due to vasospasm. Patient 4 developed Broca's aphasia and right hemiplegia. The patient was discharged from the hospital with an mRS score of 2, which improved to 1 without aneurysm recurrence at 28 months visit. Furthermore, patient 8 developed transient hemiparesis. After the symptomatic therapy, no neurological deficits were observed at discharge.

Follow-up angiography after the endovascular procedure was available for 12 patients with an average of 7.1 months (range 3–30 months). Overall, 11 of 12 BBAs (91.2%) had Raymond grade I, and only one patient (Patient 2) demonstrated neck residue (Raymond II). Two angiographic results from another hospital showed no progression in the residue. The patient maintained an mRS score of 0 at the 34-month postoperative follow-up. Additionally, the imaging results revealed stent shortening in four patients (Patients 1, 4, 8, and 10). Neurological outcomes were available for all 12 patients. Ten patients had an mRS score of 0 at discharge and at the latest clinical follow-up. Although Patient 4 sustained motor aphasia and hemiplegia, her mRS score increased from 2 at discharge to 1 at 28 months follow-up. The mRS score of Patient 1 remained at 1 from discharge to the last follow-up. The mean follow-up was 25.3 months (range, 11–36 months). Descriptions of the periprocedural and follow-up information are summarized in Table 2.

**Table 2:** Summary of clinical and angiographic outcomes of patients treated by PED-assisted coil embolization.

**Case no**.	**PED size**	**Treatment**	**Im-DSA**	**Intra- complication**	**Post- complication**	**Follow-up angiography**	**PED shortening (Y/N)**	**mRS at Discharge**	**mRS at latest FU**	**Latest FU time (mos)**
1	PED-375-20	PED + coil	1	Rupture, vasospasm	Left hemiparesis	CO (6 months)	Y	1	1	36
2	PED-475-20	PED + coil	1	N	N	NR^*^(30 months)	N	0	0	34
3	PED-325-30	PED + coil	1	Vasospasm	N	CO (5 months)	N	0	0	30
4	PED-300-18	PED + coil	1	Vasospasm	Right hemiplegia; Broca aphasia;	CO (3 months)	Y	2	1	28
5	PED-500-20	PED + coil	1	N	N	CO (3 months)	N	0	0	27
6	PED-400-16	PED + coil	1	N	N	CO (10 months)	N	0	0	21
7	PED-350-18	PED + coil	1	N	N	CO (3 months)	N	0	0	18
8	PED-475-20	PED + coil	1	Vasospasm	Transient hemiparesis	CO (6 months)	Y	0	0	11
9	PED-475-20	PED + coil	1	N	N	CO (3 months)	N	0	0	25
10	PED-400-16	PED + coil	1	Vasospasm	N	CO (6 months)	Y	0	0	31
11	PED-375-20	PED + coil	1	N	N	CO (4 months)	N	0	0	14
12	PED-325-30	PED + coil	1	N	N	CO (6 months)	N	0	0	29

*PED, Pipeline embolization device; Intra, Intraoperative complication; Post, post operation complication; mRS, modified Rankin Scale; RS, Raymond grade; Im, immediate; FU, follow-up; CO, complete occlusion; NR, neck remnant*.

* *The latest angiography showed no progression of the residue*.

### Typical Case

**Figure 1:**
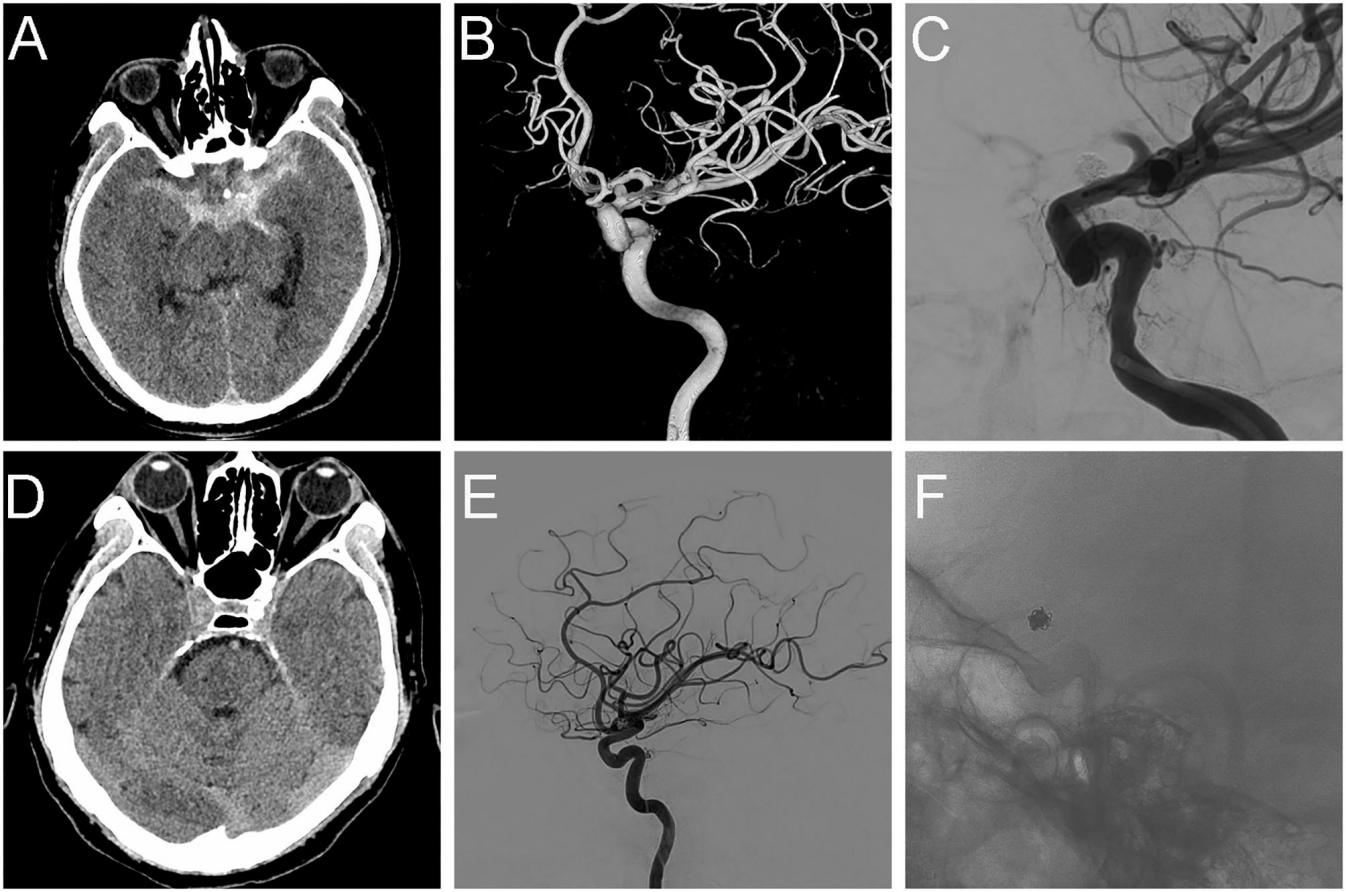
Typical case images. **(A)** CT scan showing SAH in suprasellar cistern before operation. **(B)** Preoperative DSA showing BBA at C7 segment of the left ICA. **(C)** Intraoperative DSA showing complete occlusion of the BBA using PED+ coils. **(D)** CT scan at 1-month follow-up. **(E,F)** DSA and Xper-CT at 6 months follow-up.

Patient 8: A 44-year-old man was admitted to our hospital with a severe headache (Hunt-Hess grade 2). Brain CT revealed diffuse SAH in the suprasellar cistern (Fisher grade 2) (Figure 1A). One day after the onset, diagnostic DSA confirmed a BBA (3.4 × 3.0 mm) in the lateral wall of the supraclinoid left ICA (Figure 1B). After two days, one-stage PED-assisted coil embolization was performed. During the operation, we selected an appropriate PED (4.75 × 20 mm) according to the diameter of the proximal ICA. After placing the Marksman microcatheter, an Echelon-10 coil embolization microcatheter was inserted into the aneurysmal sac. After filling the aneurysmal sac, we loosely covered the neck with some loops of the coil (2.5 mm × 5 cm) bulging into the ICA and fully deployed the PED to compact the coil. The DSA performed immediately revealed a complete obliteration (Figure 1C). The patient experienced transient hemiparesis due to vasospasm after surgery. There were no neurological deficits at discharge after symptomatic treatment. He was discharged from the hospital four days later and continued to receive dual antiplatelet therapy. The CT scan was reviewed one month later and showed that the SAH had been fully absorbed (Figure 1D). Follow-up angiography at 6 months revealed that the BBA was occluded without stenosis of the ICA. However, we found that the distal end of the PED moved toward the proximal part of the parent artery and retained complete coverage of the aneurysmal neck (Figures 1E,F). The patient had no neurological deficits at discharge or at the latest follow-up (mRS 0).

## Discussion

Blood blister aneurysms are complex and challenging vascular lesions that lack an identifiable neck, and they have a high risk of rupture (9). Owing to the intricate histopathological features and urgent SAH manifestations, aggressive treatment must be adopted once diagnosed. Although the efficacy of microsurgical, interventional, and combined methods for BBAs have been demonstrated, no one-fit-all solution exists (10–16). Successful microsurgical treatment of BBA has been demonstrated, including clipping, wrap-clipping, suturing, and extracranial-intracranial bypass (4, 17). Wrap-clipping, in comparison to wrapping and clipping, has been shown in several studies with low recurrence and re-rupture rates (18, 19). However, unbefitting clip-wrapping had a negative effect on therapeutic effect, resulting in arterial stenosis, perforator injury, and ischemic consequences (13, 20). Thus, microsuturing may be able to resolve the issue of ICA stenosis, and some literature has documented surprising results (14, 21). Significantly, the fact that technical requirements, complications at the suture site, and atherosclerosis in some cases may influence the results of microsuturing. Besides, extracranial-intracranial bypass is also a prospective approach for ruptured BBAs. Balik et al. revealed in a meta-analysis that the bypass strategy is superior to non-bypass in terms of safety and efficacy (22). In addition, the ischemic risk associated with BBAs treated with extracranial-intracranial bypass, as well as the timing and selection of procedure, should be rationally assessed.

With the introduction of various intracranial stents, endovascular therapy has gradually become the primary strategy in the treatment of BBA (19). The treatment paradigm has changed from simple coil embolization to guiding blood flow away from the aneurysm and reconstructing the parent artery. In recent years, the use of Willis covered stents for BBA treatment has yielded promising results (23, 24). However, the difficulty in delivery, endoleakage, and occlusion of branches limit its application. While stent-assisted coil embolization demonstrated high rates of immediate occlusion, the final obliteration was indistinguishable from other endovascular treatment techniques (25, 26). In addition, bleeding recurrence due to insufficient flow diversion raised concerns about this technique (27). Therefore, the presence of multiple overlapping stents combined with coiling embolization contributes to a significant reduction in bleeding recurrence and an increase in aneurysm occlusion rates (26, 28, 29). In a multicenter study of 221 patients, Fang et al. demonstrated that the complete occlusion rates of BBAs treated with a single stent, two stents, and three or more stents were 42.9%, 78.4%, and 88.2%, respectively. Furthermore, the recurrence rates were 38.1%, 13.5%, and 5.9%, respectively (30). However, deploying a second stent is not always feasible because of the procedure complexity.

The introduction of FDs represents a paradigm shift in therapeutic approaches from intra-sac embolization to vessel reconstruction (31). This strategy has proven to have a lower retreatment rate than microsurgery and other endovascular treatment (18). As the most commonly used FD, PED has been approved to treat unruptured aneurysms. Nonetheless, its application in ruptured aneurysms is limited owing to delayed occlusion, with only a few reports of ruptured BBAs being treated. In this study, all patients with BBAs underwent single PED-assisted coil embolization. During angiographic follow-up, 12 patients achieved complete embolization, but one developed a neck residue. During a mean follow-up of 25.3 months, 12 patients maintained good neurological function (mRS score ≤ 2). Accordingly, we demonstrated that PED-assisted coil embolization is a safe and effective strategy for BBA treatment.

There is controversy over using a coil in conjunction with PED for BBA treatment. In a multicenter study of 45 patients, Mokin et al. demonstrated that a single PED is a safe and effective modality, and the use of coils is not associated with eventual occlusion (7). Besides, several small sample studies have also reported success with PED alone in the treatment of BBAs (11, 32, 33). Overall, these studies indicate that a single PED may be sufficient for BBA treatment. However, incomplete or delayed occlusion and persistent aneurysm growth during thrombosis in the aneurysm sac have increased, which is uncommon in traditional endovascular procedures. Ryan et al. reported 16 BBAs; none of the aneurysms was completely occluded on the day of treatment, and only 31% were completely occluded at three months follow-up (34). Some studies have also reported aneurysm growth and catastrophic re-rupture following a single PED deployment, leading to death (6, 35, 36). In addition, although intraoperative contrast stasis and decreased blood flow were observed in the aneurysm sac after PED placement, this finding did not suggest a decrease in pressure. Computational hemodynamic research conducted by Cebral et al. (37) illustrated that increased intra-aneurysmal pressure after FD deployment was associated with re-rupture.

These cases indicate that early intravascular embolization may be a reasonable strategy for avoiding re-rupture. Therefore, some studies advocate for a therapeutic strategy using coil embolization followed by PED to re-establish blood flow in the parent artery (6, 10, 38, 39). The coil was used to embolize the aneurysm cavity, loosely pack the aneurysm neck, and assist the PED in repairing the parent artery. Thus, the parent artery was reconstructed simultaneously with the embolization of the aneurysm. Our patient showed favorable outcomes. Immediate postoperative angiography revealed that all BBAs were successfully achieved one-stage embolization. However, this modality involves intraluminal surgery, which theoretically increases the risk of intraoperative aneurysm rupture. Contrast leakage occurred during coil packing in Patient 1, and re-rupture of the aneurysm was contemplated. The aneurysmal sac was rapidly filled with coils, and the PED was opened without any leakage. Based on this experience, we selected coils smaller than the measured diameter and adopted a more cautious approach to reduce the probability of intraoperative rupture in other patients.

The stability and shape of the microcatheter as well as the coiling microcatheter delivery timing into the aneurysm cavity are critical for this surgery. The PED deployment may cause the fluctuation of the coaxial system, which may cause the coiling microcatheter to move. The coiling microcatheter could either enter the aneurysm cavity so as to rupture the aneurysm or drop out of the aneurysm cavity and thus extending the operating time and increasing the risk of stent thrombosis. Furthermore, the time of the PED in the semi-deployed state should be reduced to prevent stent thrombosis. The PEDs were successfully deployed in all the patients in our study. In Patient 2, the PED was failed to be fully expanded despite the wire being swayed gently. Finally, the PED was fully opened with the assistance of a balloon. In addition to these two operations, re-deployment following PED recycling is an effective solution to solve issues, such as stent malposition. All the patients achieved complete obliteration at the follow-up angiography, except one patient with slight neck residue. It may be associated with the formation of an intractable wedge-shaped space between the PED and aneurysm neck. At the 7 and 30-month angiography follow-up, two angiographic results from a different hospital demonstrated no progress in the residue. At the 34-month clinical follow-up, this patient maintained an mRS score of 0. Therefore, further invasive therapy was not necessary because of the expected low risk of re-bleeding from such a minimal residue. If progression is observed during subsequent imaging examination, then a second PED may be considered.

In our center, we also adopted the technique of “endovascular patch embolization” to repair the fragile neck, which is one of the challenges in BBA therapy (40). The advantage of this technique is that embolization of the aneurysm neck can be achieved using coils and staged-deployed PED. After filling the aneurysmal sac, we used a few loops of coil herniation into the parent vessel to loosely cover the neck and fully deploy PED to compact the coil. Our practice has proved the safety and efficacy of this patching technique.

Imaging follow-up also revealed stent shortening in four patients, which was thought to be a key factor influencing treatment success. Wang et al. indicated that this is relative stent shortening due to changes in the parent vasculature during BBA formation. After PED deployment, the shortened parent arteries reverted to their original length, resulting in the appearance of a shorter PED. However, this does not preclude the possibility of stent shortening, as the PED is entirely expanded (41). A review of our intraoperative findings of vasospasm in these four patients supports this viewpoint. Notably, this “shortening” did not cause aneurysmal recanalization. Accurate assessment of the aneurysm and parent artery, proper PED size, and adequate anchoring length around the BBA are crucial for preventing stent migration and shortening. The size of PED used was selected based on the diameters of the ipsilateral distal and proximal non-vasospasm segments or the relatively normal contralateral artery.

Because the BBA is typically located in the supraclinoid of the ICA, occlusion of the branch vessels is unavoidable, causing ischemic complications. However, despite coverage of the ICA branches and anterior cerebral artery, no associated problems were detected in this study. In their meta-analysis, Cagnazzo et al. included 757 ophthalmic arteries, and 196 posterior communicating arteries demonstrated that none of the patients had symptoms related to decreased blood flow or occlusion. In 199 prechoroidal arteries, the pooled results indicated that only 1% of patients experienced transient neurological symptoms (42). Bhogal et al. analyzed 147 aneurysms treated with FDs and illustrated that the side-branch occlusion rate of the ICA was 20%. However, none of the patients showed clinical symptoms of arterial occlusion (43). Although FD covers the collateral vessels, the strong collateral circulation system may explain lower occlusion rates and rare manifestations. Therefore, we do not have to worry about branch patency when using FD.

Currently, there is no consensus on the timing and duration of antiplatelet therapy using PED to treat ruptured BBA (34, 39, 44). An ideal protocol should effectively prevent in-stent thrombosis while minimizing the risk of drug-related bleeding. Combining ASA and clopidogrel has become the most commonly used strategy, despite the different regimes. However, in five cases of ruptured BBAs treated *via* PED, Tanburoglu et al. demonstrated that single antiplatelet therapy is safe and effective (45). In this study, antiplatelet treatment was not administered before the procedure. The protocol for promptly administering tirofiban and bridging loading doses of aspirin and clopidogrel was successful. No ischemic or hemorrhagic complications were associated with antiplatelet therapy during the perioperative period. This demonstrated the safety and efficacy of this modified strategy. This favorable outcome may be explained by the decreased risk of re-rupture caused by coiling embolization, and the modified antiplatelet strategy used to avoid thrombosis. Saber et al. (46) reviewed 2,002 patients treated with PED and found performing platelet function testing (PFT) did not 229 predict hemorrhagic and ischemic events. Besides, some studies have demonstrated that patients undergoing PFT have an increased risk of hemorrhagic complications (47, 48). Therefore, PFT was not performed at our center. In summary, additional prospective studies on antiplatelet strategies after PED placement are needed.

### Limitation

Limitations of this study include its retrospective design, single-center sample size, lack of a defined control group, and a small number of patients involved (12 patients). All of which may have contributed to the selection and reporting bias. Given the rarity of BBAs and the scarcity of reports on PED-assisted coil embolization, this study provides strong evidence for advocating this technique.

## Conclusion

The favorable outcomes from this study suggest that PED in conjunction with coils is safe and efficacious in the treatment of ruptured BBAs. It has enhanced our understanding of BBA therapy and provided a potentially optimal option for this intractable lesion. However, further evidence from prospective and multicenter studies is necessary.

## Data Availability Statement

The original contributions presented in the study are included in the article/supplementary material, further inquiries can be directed to the corresponding author/s.

## Author Contributions

LL, CW, and XX contributed to the study design. PL, SL, and TW performed the clinical follow-up. PL and LL performed the literature search, data collection, and drafted the manuscript. LZ, TW, XX, and CZ contributed to data analysis and interpretation. XX, SL, and CW contributed to editing and revision of the manuscript. All authors read, edited, and approved the final version of the manuscript.

## Funding

This work is supported by the Research and Development Projects in Sichuan Province (Grant 2021YFS0204).

## Conflict of Interest

The authors declare that the research was conducted in the absence of any commercial or financial relationships that could be construed as a potential conflict of interest.

## Publisher's Note

All claims expressed in this article are solely those of the authors and do not necessarily represent those of their affiliated organizations, or those of the publisher, the editors and the reviewers. Any product that may be evaluated in this article, or claim that may be made by its manufacturer, is not guaranteed or endorsed by the publisher.

## Abbreviations

BBA: blood blister aneurysm
PED: pipeline embolization device
FD: flow diverter
3D: three-dimensional
DSA: digital subtraction angiography
mRS: modified Rankin Score
PFT: platelet function testing.
